# “When you see a fork in the road, take it”—*Yogi Berra*

**DOI:** 10.1007/s12471-023-01801-3

**Published:** 2023-07-24

**Authors:** Yigal M. Pinto, Martin E. W. Hemels

**Affiliations:** 1 Department of Experimental Cardiology, Amsterdam University Medical Centres, location Academic Medical Centre, University of Amsterdam, Amsterdam, The Netherlands; 2 Department of Cardiology, Rijnstate Hospital, Arnhem, The Netherlands; 3 Department of Cardiology, Radboud University Medical Centre, Nijmegen, The Netherlands

This special issue of the *Netherlands Heart Journal* is dedicated to the clinical and scientific career of Professor Arthur Wilde, one of the giants of cardiology. While his retirement in August of this year inevitably necessitates to look back, his work and its impact in the field hold a number of important messages that actually concern the future of cardiology. As such, the career of Professor Wilde can be viewed as a clear example of how to connect curiosity-driven basic research to clinical implications, and by doing so, the art of cardiology itself and—most importantly—the treatment and outcomes for patients with heart disease can be changed.

Wilde started his career in the Experimental Cardiology department headed by Professor Michiel Janse, where he was involved in pre-clinical electrophysiological studies. While being trained as a clinical cardiologist, he remained strongly connected to this pre-clinical work and later succeeded Professor Janse to head the same department, before he became the head of the Clinical Cardiology department at the Amsterdam University Medical Centre. As Yogi Berra once wrote, “When you see a fork in the road, take it“ [1]. By taking this fork in the road, Wilde was able to connect pre-clinical and clinical electrophysiology. Understanding the very basic underpinnings of the electrocardiogram (ECG) undoubtedly added to his internationally acclaimed skill to analyse and interpret ECGs, demonstrating the enormous added value such profound ability has for daily patient care. This ability became quickly evident in his work on often complicated and subtle changes in patients with long QT syndrome. Again, he took the fork in the road by combining meticulous clinical assessments of these patients with pre-clinical mimicking of channel properties in cultured cells. This eventually led to several breakthrough findings that have sparked the field of cardiogenetics, where care of patients with inherited arrhythmias is fuelled by a deep basic understanding of the underlying molecular mechanism. As a pioneer of this new field, he set up cardiogenetic counselling, which is now an integral part of cardiology practice and changes the lives of the many patients affected.

Looking at the immense impact his work has had in the field, which lessons can be drawn? In this special issue of our journal, we aim to answer this question by highlighting different topics related to Wilde’s scientific work. In a review, Peltenburg and colleagues describe the current knowledge gaps in the 3 most common inherited arrhythmia syndromes: Brugada syndrome, congenital long QT syndrome and catecholaminergic polymorphic ventricular tachycardia [2]. Although a huge amount of knowledge on both the diagnosis and treatment of these syndromes has been gathered in the last decades, important steps still have to be taken in early recognition and identification of patients who are at greatest risk. The authors therefore state that continuous collaborations between clinicians and pre-clinical researchers worldwide remains of the utmost importance.

Although a genetic substrate has not been identified in a majority of patients with idiopathic ventricular fibrillation (IVF), a founder risk haplotype—located on chromosome 7q36 and comprising the gene for dipeptidyl aminopeptidase–like protein 6 (*DPP6*)—was identified in the Netherlands and published in 2009 [3]. In this issue, Bergeman and colleagues describe the long-term follow-up of the Dutch IVF DPP6 risk haplotype cohort and provide important new insights into the natural history of the condition and the value of implantable cardioverter-defibrillator therapy using current risk stratification [4]. A different unique gene variant found in the Netherlands many years ago is *SCN5A*-1795insD, which causes an overlap syndrome encompassing features of both loss and gain of sodium channel function. Proost et al. composed a historical overview describing the journey and results of 7 decades of translational research on this founder variant [5].

Different types of cardiomyopathy also have a genetic basis. The article by Bos et al. describes the characteristics of different genetic variants associated with the arrhythmogenic cardiomyopathy phenotype, which is characterised by life-threatening ventricular arrhythmias and heart failure [6]. Schoonvelde and colleagues identified 2 female patients with desmoplakin cardiomyopathy, an inherited cardiomyopathy presenting with recurrent episodes of acute myocardial injury [7]. The authors state that cardiac magnetic resonance imaging is key in the diagnosis of desmoplakin cardiomyopathy due to its specifying imaging features. The pathogenic p.(Arg14del) variant in the *PLN* gene also results in a high risk of developing arrhythmogenic or dilated cardiomyopathy with heart failure. In a high-level discussion between 2 experts in this field, De Boer and Doevendans analyse the pros and cons of offering preventive treatment to all carriers of this variant [8, 9]. In addition, Van Lint and coworkers discuss whether exercise influences the development of an arrhythmic phenotype in *PLN* p.(Arg14del) cardiomyopathy [10]. Finally, a retrospective cohort of patients with the pathogenic founder variant *MYH7* p.(Arg1712Gln) and a consistent hypertrophic cardiomyopathy phenotype is described by Marsili et al. [11]. Given the fact that these patients may present with delayed penetrance, it is suggested that clinical follow-up should be pursued after the seventh decade in healthy carriers and that longer intervals between screening may be justified in healthy women younger than 30 years.

The abovementioned papers illustrate once more how medicine always—without exception—benefits from a deeper understanding of disease mechanisms and that when doctors are able to connect deep-diving experimental approaches to meticulous clinical care, they are able to implement such deeper understanding to change therapy for the patients they treat. This cycle from curiosity towards patient care and back has been the engine that drives innovation across medicine. Role models such as Arthur Wilde have shown that this century-old tried and tested engine of innovating our ideas will never cease to have great impact on the patients we see on a daily basis.
